# Validation of the generalized anxiety disorder screener (GAD-7) in Cypriot pregnant and postpartum women

**DOI:** 10.1186/s12884-022-05127-7

**Published:** 2022-11-15

**Authors:** Paris Vogazianos, Emma Motrico, Sara Domínguez-Salas, Andri Christoforou, Eleni Hadjigeorgiou

**Affiliations:** 1 Department of Social and Behavioral Sciences, School of Humanities, Social and Education Sciences, European University Cyprus, Nicosia, Cyprus; 2 Psychology Department, Universidad Loyola Andalucia, Seville, Spain; 3 Nursing Department, School of Health Sciences, Cyprus University of Technology, Nicosia, Cyprus

**Keywords:** GAD-7, Anxiety, Perinatal anxiety, Pregnancy, Postpartum

## Abstract

**Background:**

Anxiety is increasingly acknowledged as a common mental health issue during the perinatal period. Its prevalence as well as the associated adverse effects constitute screening imperative. This study evaluates the psychometric properties and underlying factor structures of a Greek version of GAD-7 among pregnant and postpartum women (up to 6 months) in Cyprus.

**Methods:**

This study was conducted from June to December 2020. A total of 457 Cypriot women in the perinatal period (222 pregnant and 235 postpartum) were surveyed. The assessment included anxiety (GAD-7) and depression (EPDS), and psychosocial factors related with anxiety. The internal consistency and factor structure of GAD-7 were evaluated using reliability coefficients, Cronbach’s Alpha and McDonald's Omega, and factor analysis, both Exploratory as well as Confirmatory.

**Results:**

GAD-7 demonstrated good internal consistency (α = 0.907; Ω = 0.909). Horn's parallel analysis indicated a single factor as the most appropriate. CFA using the standard ML method indicated a good model fit, χ^2^ = 21.207, *p* = 0.096; CFI = 0.999; SRMR = 0.027. More studies are needed to determinate the cut-off point and the maximisation of the scale’s sensitivity and specificity in pregnant and postpartum Greek Cypriot women.

**Conclusions:**

GAD-7 is a valid and reliable measure and healthcare professionals should utilize GAD-7 as a standard instrument for the screening of anxiety symptoms in pregnant and postpartum Greek Cypriot women.

## Background

Perinatal anxiety has been receiving growing attention in the literature in recent years. A systematic review and meta-analysis of 102 studies with 221,974 women from 34 countries estimated that the prevalence for self-reported anxiety symptoms was 18.2% in the first trimester, 19.1% in the second trimester, 24.6% in the third trimester, and 15% at 1–24 weeks postpartum [1]. A recent meta-analysis by Fawcett et al. [2] suggests that 1 in 5 women in the perinatal period meet the diagnostic criteria for at least one anxiety disorder, constituting anxiety disorders with perinatal onset much more prevalent than previously thought. While significant differences in study methodology result in a wide variation in the estimates of anxiety-related disorders or symptoms [3, 4], it is now well-established that the prevalence of anxiety warrants clinical attention [1, 2, 5]. In addition, the association of perinatal anxiety with adverse consequences on birth and neonatal outcomes [6–8], mother-infant relationship [9, 10], as well as the association with postpartum depression [11, 12] constitute screening imperative.

The National Institute for Health and Care Excellence [13] states that the range and prevalence of anxiety disorders are under-recognised both during pregnancy and the postpartum period. The American College of Obstetricians and Gynecologists [14] recommends that women in the perinatal period are screened at least once for depression and anxiety symptoms through standardized, validated tools. More than ever, routine screening for anxiety, depression, and other mental health issues becomes of utmost importance under the current circumstances of COVID-19 pandemic [15, 16]. Proactive measures could result in prevention, early detection, and prompt referral and treatment [17].

The Generalized Anxiety Disorder-7 (GAD-7), developed by Spitzer et al. [18], is one of the recommended screening measures for perinatal anxiety by NICE [13], although it is recommended for further assessment in case a woman scores 3 or more on the GAD-2, a distinct subscale comprising of the first two GAD-7 items, whose usefulness in maternity services has been debated [19]. A recent study by Fairbrother et al. [20] showed that GAD-7 outperformed the GAD-2 in their sample of 310 Canadian at 3-months postpartum. The GAD-7 has been found to have good psychometric properties among other populations in the perinatal period such as English-speaking perinatal women in Canada [21], Spanish-speaking pregnant Peruvian women [22], Spanish-speaking pregnant women in urban Spain [23], and pregnant Chinese women in mainland China [24].

The aim of the present article is to examine the psychometric properties and the factor structure of the GAD-7 in a sample of Cypriot pregnant and postpartum women. So far, there are no published studies of the properties of the GAD-7 in Cypriot women in the perinatal period.

## Methods

### Study design

Our sample consisted of 457 Cypriot women in the perinatal period (222 pregnant and 235 postpartum) who were recruited in the framework of the international study “Impact of the Covid-19 pandemic on perinatal mental health (Riseup-PPD-COVID-19)” (Identifier: NCT04595123) [25]. The data presented here are extracted from the baseline assessment, which was conducted in the period between July 2020 and January 2021. The inclusion criteria at baseline were: (i) Pregnant or biological mother of a child six months old or younger; (ii) 18 years of age or older and (iii) Living in Cyprus. The study was conducted according to the principles expressed in the Declaration of Helsinki, whereas ethical approval for the data collection in Cyprus was obtained by the Cyprus National Bioethics Committee (ΕΕΒΚ ΕΠ 2020.01.Ι26). Electronic informed consent was obtained from all the participants, and the confidentiality of all information provided was ensured. Handling of the study data complies with all the national required standards for data protection.

### Study sample and procedure

Participants were recruited mainly through social media (Facebook, Instagram), through local organizations and maternity units in both public and private hospitals, as well as through personal networks of colleagues and acquaintances of the research team members. Data collection was implemented through Qualtrics®. Women who fulfilled the inclusion criteria were asked to click on the project website link (https://momsduringcovid.org/cyprus) so that they would be directed to the online questionnaire for Cyprus. After an overview of the study, participants were asked to confirm the set of eligibility criteria and to provide their consent to access the study. The Informed Consent provided a clear explanation of how anonymity, confidentiality and protection of data would be safeguarded. A debriefing procedure was also in place, where a list of relevant up-to-date psychosocial services and resources in Cyprus was provided to the participants at the end of the survey.

The questionnaire, which consisted of several different scales as described in Motrico et al. [25], was available in both Greek and English languages as the study targeted all women living in Cyprus with the characteristics mentioned above. The Greek translation of GAD-7 was performed following the three steps proposed by Fenn et al. [26]: (a) forward translation by at least two translators working separately (b) backward translation by at least two translators working separately, (c) check by committee of experts. The English GAD-7 was initially translated in Greek by two independent experts who were fluent in the original and target languages and cultures – one language expert and one subject expert – who worked separately and produced two translations. The two versions were compared and the minor discrepancies found were discussed and resolved. The agreed Greek version was then translated back into English by a competent language expert and a subject expert who were not involved in the forward translation. After the research team ensured that the backward translation matched the original English version and a consensus was reached to ensure that the final Greek version was equivalent in language and meaning to the English version, the measure, along with the rest of the questionnaire, was pilot tested to ensure adaptability the Greek-speaking Cypriot population.

Based on the diagnostic criteria for generalized anxiety disorder as established in the 4^th^ edition of the Diagnostic and Statistical Manual of Mental Disorders [27], the GAD-7 is a seven item self-report measure asking about core generalized anxiety disorder symptoms experienced in the previous two weeks. The measure comprises of the following items: 1. Feeling nervous, anxious, or on edge, 2. Not being able to stop or control worrying, 3. Worrying too much about different things, 4. Trouble relaxing, 5. Being so restless that it is hard to sit still, 6. Becoming easily annoyed or irritable, 7. Feeling afraid, as if something awful might happen [18]. Each item is rated on a four-point scale (0 = Not at all and 3 = Nearly every day) and total scores range from 0 to 21 [18]. To the best of our knowledge, this is the first study that investigates the psychometric properties and factor structure of the GAD-7 in a Greek-speaking sample of Cypriot women in the perinatal period.

After the completion of the baseline assessment, survey data were manually checked for accuracy and consistency before analysis. From an initial number of 1172 respondents, 477 invalid records were identified and removed because either the participant indicated erroneous pregnancy duration or their baby was over 6 months. The data of women who completed the Greek version but were not of Cypriot origin or had not specified origin (N = 238) were excluded from this analysis. The sample consisted of a total of 457 women who completed the Greek version of the questionnaire and who were of Cypriot origin. The statistical package SPSS v 26.0 was used for the analysis of data.

### Statistical analysis

For the sample size calculation using the calculator of Moshagen and Erdfelder [28] with statistical power of 95%, a significance level of 5% and an RMSEA of 0.08 we calculate the sample size a priori as n = 305. Inferential statistical analysis was performed using a significance level of 95%. All numerical variables were described using means ($\underline{x}$), medians (δ) and standard deviations (s) whereas all categorical variables were described using frequencies (f) and proportions (%). The distribution of all numerical variables was investigated through the Kolmogorov Smirnov test of Normality using R and since normality was rejected (*p* < 0.001) the differences between independent groups was examined through Mann Whitney test of independent groups using R. The internal consistency of GAD-7 was evaluated using both Cronbach’s Alpha as well as McDonalds’ Ordinal Omega, due to the fact that the items differ in quality, have different factor loadings, have positively skewed and leptokurtic distributions (Table 2) as well as have medium to low correlations between them (Table 4), [29, 30] using SPSS version 26 with Hayes Omega Macro [31] and through Polychoric Correlations of the items between themselves [32], calculated using the ‘Polycor’ library in R. The associations between GAD-7 and other variables – namely level of stress related to the COVID-19 outbreak, depression symptoms, and PTSD symptoms – were examined using Spearman’s correlation and Polychoric Correlations calculated using the ‘Polycor’ library in R. Specifically, the variables examined for associations were: Overall level of stress related to the COVID-19 outbreak (COPE-IS: Coronavirus Perinatal Experiences—Impact Survey); EPDS: Edinburgh Postnatal Depression Scale; 10 questions from PTSD Checklist for DSM-5 asking participants to indicate the extent to which they experienced the following: (1) Feeling super alert or watchful or on guard; (2) Feeling jumpy or easily startled; (3) Having difficulty concentrating; (4) Trouble experiencing positive feelings; (5) Feeling guilty or blaming yourself; (6) Feeling irritable, angry or aggressive; (7) Repeated disturbing and unwanted thoughts about the COVID-19 outbreak; (8) Repeated disturbing dreams about the COVID-19 outbreak; (9) Trying to avoid information or reminders about the COVID-19 outbreak; and (10) Taking too many risks or doing things that could cause you harm, whereas the total PTSD score was calculated by summing the 10 items [25].

**Fig. 1:**
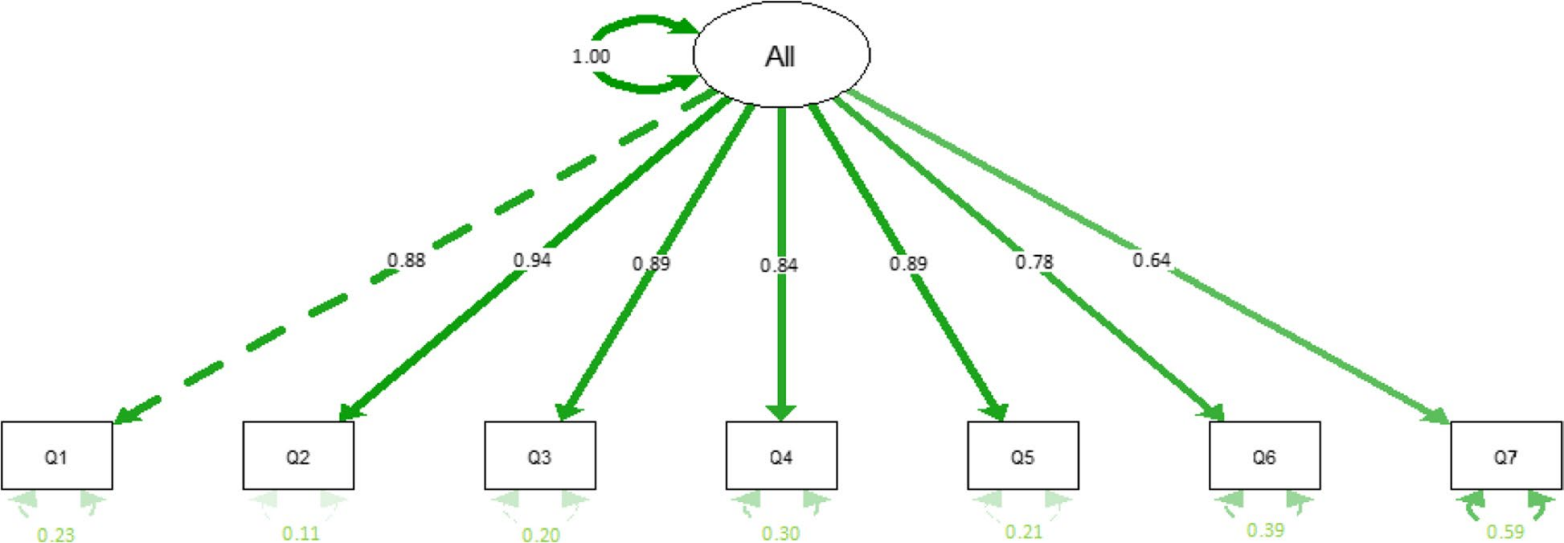
CFA Path Diagram

The association of GAD-7 and receiving treatment for mental health concerns was examined through Mann Whitney test of independent groups using R. The factor structure of GAD-7 was examined through Horn's Parallel Analysis for factor retention [33] calculated using the ‘Paran’ library in R, and Confirmatory Factor Analyses (CFA) was calculated using SPSS version 26. Since the standard Maximum Likelihood (ML) method in the CFA analysis assumes a continuous and multivariate normal distribution, the Robust Maximum Likelihood (MLR) was also used in case this normality assumption was violated [34]. In the CFA analysis, to correctly evaluate the fit of a model, we used multiple indexes [35–39] such as the model chi-square (χ^2^), the model standardised chi-square (χ^2^/df), the Comparative Fit Index (CFI), the Root Means Square Error of Approximation (RMSEA), the Standardized Root Mean Square Residual (SRMR) and the Tucker-Lewis Index (TLI). The cut-off points, for the fit indices, for a good fit according to Hu, & Bentler, [40] are CFI ≥ 0.95; TLI ≥ 0.95; SRMR ≤ 0.08; RMSEA ≤ 0.06 whereas according to Browne & Cudeck, [41] and MacCallum, Browne & Sugawara, [42], 0.06 ≤ RMSEA ≤ 0.1 suggest an adequate fit. Finally, according to Cole, [43] the χ^2^, and χ^2^/df should not be significant. Fig. 1.

## Results

The study sample consisted of 222 pregnant and 235 postpartum women (with infants up to 6 months of age). For 60.6% of the participants, this was their first pregnancy (59.5% of pregnant women and 61.7% of postpartum women). The rest of demographic characteristics of the sample are described in Table 1.

**Table 1:** Demographic characteristics

		N	%
Age	18–25	9	2.0
	26–35	311	68.1
	36–45	111	24.3
	Did not respond	26	5.7
Highest level of education	Secondary school/ High school	21	4.6
	Partial university studies	26	5.5
	University studies (undergraduate)	173	37.9
	Master or Doctorate	234	51.2
	Other	3	0.7
Relationship status	Single	2	0.4
	Partnered/engaged or living as a couple	77	16.8
	Married	375	82.1
	Separated or divorced	3	0.7
Residence	Owned (paid in full)	200	43.8
	Owned (paying mortgage)	101	22.1
	Rented	107	23.4
	Living with parents	31	6.8
	Living with others	9	1.9
	Other	9	2.0

Individual item means, standard deviations, item-total correlations, and Cronbach’s alphas with items removed are presented in Table 2. The Kolmogorov Smirnov test of Normality indicated a lack of Normality in the overall GAD-7 (*p* < 0.001) whereas the Mann Witney test of independent groups indicated non-statistically significant tests between pregnant women and new mothers (*p* = 0.291).

**Table 2:** GAD-7 Item means, medians, standard deviations, skewness, kurtosis, corrected item-total correlations (r), Cronbach’s alpha with item removed (α), factor loadings, and communalities

Item	Q1	Q2	Q3	Q4	Q5	Q6	Q7
Mean	0.71	0.50	0.84	0.89	0.43	0.84	0.56
Median	0.00	0.00	1.00	1.00	0.00	1.00	0.00
SD	0.88	0.78	0.86	0.91	0.76	0.88	0.79
Skewness	1.19	1.57	0.95	0.88	1.88	1.01	1.53
Kurtosis	0.69	1.81	0.38	0.06	3.11	0.47	2.00
r	0.76	0.81	0.79	0.74	0.75	0.68	0.54
α	0.89	0.88	0.89	0.89	0.89	0.90	0.91
Factor loading	0.82	0.87	0.83	0.78	0.79	0.72	0.56
Communalities	0.66	0.76	0.68	0.60	0.63	0.51	0.32

The internal consistency of the GAD-7 was high (Cronbach’s Alpha = 0.907 and MacDonald’s Ordinal Omega = 0.909). An examination of the response categories of the seven items on the GAD-7 indicate that results were mainly distributed in the lowest answer categories for each item. There was mainly a tendency towards a floor effect in questions 5 (Intense nervousness, to the point that you cannot stay calm) and 2 (Inability to calm down or control your worries), with an 69.1% and a 64.8% accumulation of answers on the lowest rating (“not at all”). All the items’ percentages of respondents for each answer in each item are shown on Table 3. A floor effect was also available in items 1 (Nervousness, anxiety or agitation) and 7 (Fear that something tragic will happen) as indicated by the median answers (δ = 0).

**Table 3:** Percentages of respondents for each answer in each item of GAD-7

Item	Q1	Q2	Q3	Q4	Q5	Q6	Q7
0	51.0	64.8	39.8	38.5	69.1	39.8	58.6
1	33.9	24.1	43.3	41.8	22.1	44.0	31.7
2	8.5	7.9	10.3	11.4	5.0	8.3	5.0
3	6.6	3.3	6.6	8.3	3.7	7.9	4.6

The correlation between the GAD-7 items ranged from 0.85 to 0.47 (Table 4).

**Table 4:** Polychoric correlations of the GAD 7 scale items

	Q1	Q2	Q3	Q4	Q5	Q6	Q7
Q1	1.00						
Q2	0.85	1.00					
Q3	0.75	0.85	1.00				
Q4	0.68	0.76	0.77	1.00			
Q5	0.79	0.81	0.76	0.77	1.00		
Q6	0.72	0.71	0.66	0.67	0.72	1.00	
Q7	0.53	0.58	0.64	0.53	0.55	0.47	1.00

GAD-7 had significant correlations with all of the variables examined for construct validity (*p* < 0.001) with Polychoric correlations ranging from a maximum of 0.767 and 0.759 (with PTSD total and EPDS respectively) to a minimum of 0.326 (with PTSD9) and the Spearman’s correlations ranging from a maximum of 0.747 and 0.744 (with PTSD total and EPDS respectively) to a minimum of 0.180 (with PTSD10) (Table 5). Similarly, the participants who reported receiving treatment for mental health concerns at the time of the study had significantly (*p* = 0.009) higher levels of GAD-7 ($\underline{x}=7.56, \sigma =5.14,$ δ = 7) versus the participants not receiving treatment (median value of ($\underline{x}=4.66, \sigma =4.65,$ δ = 3).

**Table 5:** Associations between GAD-7 and other variables

	Polychoric Correlations	Spearman's Correlations	Mann Whitney U	*p* value
Receiving treatment			2181.5	0.009^#^
LEVEL OF STRESS	0.514	0.482		< 0.001^*^
EPDS	0.759	0.744		< 0.001^*^
PTSD1	0.668	0.604		< 0.001^*^
PTSD2	0.508	0.414		< 0.001^*^
PTSD3	0.660	0.581		< 0.001^*^
PTSD4	0.710	0.613		< 0.001^*^
PTSD5	0.661	0.523		< 0.001^*^
PTSD6	0.672	0.611		< 0.001^*^
PTSD7	0.513	0.466		< 0.001^*^
PTSD8	0.459	0.303		< 0.001^*^
PTSD9	0.326	0.291		< 0.001^*^
PTSD10	0.357	0.180		< 0.001^*^
PTSD Total	0.767	0.747		< 0.001^*^

^*^ Based on the Spearman’s Correlations

^#^ Based on the Mann Whitney U test of independent groups

Optimal implementation of Horn's parallel analysis was carried out, using the eigen decomposition of correlation matrix, to determine the most appropriate number of factors. The results advocate in favour of extracting a single factor because the adjusted eigen value was greater than 0 (Table 6).

**Table 6:** Optimal implementation of parallel analysis with 5000 iterations, using the mean estimate

Factor	Adjusted Eigenvalue	Unadjusted Eigenvalue	Estimated Bias
1	3.924711	4.120452	0.195741
2	-0.022972	0.094691	0.117663
3	-0.050621	0.007501	0.058123
4	-0.029775	-0.02341	0.006356
5	-0.000459	-0.04215	-0.04169
6	-0.023827	-0.11608	-0.09225
7	0.013873	-0.13773	-0.1516

*Adjusted eigenvalues* > *0 indicate dimensions to retain. (1 factor retained)*

The confirmatory factor analysis (CFA) testing using the standard maximum likelihood method and the one-factor structure indicated a good model fit, χ^2^ = 21.207, p = 0.096; χ^2^/df = 1.51, p = 0.218; CFI = 0.999; TLI = 0.999; RMSEA = 0.034, *p* = 0.818; SRMR = 0.027 (see Table 5) where all the cut off points, for a good fit, were adhered. On the other hand, the confirmatory factor analysis (CFA) testing using the robust maximum likelihood method and the one-factor structure indicated an acceptable model fit, χ^2^ = 53.899, *p* < 0.001; χ^2^/df = 3.85, *p* = 0.0497; CFI = 0.994; TLI = 0.991, RMSEA = 0.079, *p* = 0.015; SRMR = 0.027 (see Table 5) where the cut off points for CFI, TLI and SRMR suggest a good fit, RMSEA and χ^2^/df suggest an adequate fit, whereas the χ^2^ was not adhered.

## Discussion

The present study is the first to examine the psychometric properties and factor structure of the GAD-7 in a sample of Greek-speaking pregnant and postpartum Cypriot women. The internal consistency of the GAD-7 was high (Cronbach’s Alpha = 0.907 and MacDonald’s Ordinal Omega = 0.909), while the correlation between the GAD-7 items ranged from 0.85 to 0.47. Our results indicated no significant differences between pregnant (*N* = 222) and postpartum (*N* = 235) women (*p* = 0.291). Correlations between GAD-7 and overall levels of stress, depression symptoms, and PTSD symptoms were all positive and significant (*p* < 0.001), while the participants reporting receiving treatment for mental health concerns at the time of the study had significantly higher (*p* = 0.009) GAD-7 results than the rest of the participants. Horn's parallel analysis supported the extraction of a single factor as optimal.

Our findings strengthen the evidence for the use of GAD-7 as a brief screening measure for anxiety in the perinatal period [21–24, 44]. Another strength of the present study is that the results are based on women who span a spectrum of the perinatal period, namely early pregnancy until 6 months postpartum, unlike other studies which focused only on pregnancy [22, 23] or on even more specific periods, such as the first trimester [24]. As NICE’s (2014) [13] recommendations for the use of GAD-2 and GAD-7 were mainly based on expert consensus rather than research findings [19], this study fills a gap in the literature, contributing further to the validation of GAD-7 in the perinatal population. It also contributes to the debate in recent literature [20] providing evidence for the usefulness of GAD-7 in screening for anxiety during pregnancy and up to 6 months postpartum. Our study is in line with previous studies [21–23] that found high internal consistency and one-factor solution. Therefore, our results support the current international evidence favouring a one-factor structure for the GAD-7 during the perinatal period.

The major limitation of this study is the lack of data from other self-report anxiety measures and perhaps more importantly, the lack of data obtained through gold standard methodology, i.e. through well-validated diagnostic structures such as clinical interviews [22, 24]. The study design did not allow us, therefore, to utilize a cut-off point and maximize the sensitivity and specificity. Nevertheless, the study results provide evidence that GAD-7 can be used as a first screening measure of perinatal anxiety, both for prevention purposes and also for further follow ups including diagnostic interviews and interventions if warranted. It is important to highlight, however, that further validation studies in different socio-cultural contexts are needed if GAD-7 is intended for use in clinical practice with perinatal populations. As a validation study with pregnant women from Ghana and Côte d’Ivoire [45] suggests, GAD-7 may perform better in higher-income countries.

## Conclusion

In summary, the present study indicates that the excellent psychometric properties of GAD-7 for Greek Cypriot pregnant and postpartum women (up to 6 months) constitute GAD-7 an appropriate brief screener for anxiety in the perinatal period. As such, it can be incorporated into standard practice of intrapartum care in Cyprus for the prevention and treatment of anxiety as necessary. These research findings are especially relevant for clinicians and mental health professionals working with women in the perinatal period, and particularly those who are at risk of developing or having pre-existing anxiety symptoms.

## Data Availability

The datasets generated and/or analysed during the current study are not publicly available due ethical restrictions but are available from the corresponding author on reasonable request.
